# Nutritional Disorders in a Group of Children and Adolescents with Syndromes or Diseases Involving Neurodysfunction

**DOI:** 10.3390/nu13061786

**Published:** 2021-05-24

**Authors:** Justyna Podgórska-Bednarz, Lidia Perenc, Mariusz Drużbicki, Agnieszka Guzik

**Affiliations:** 1 Institute of Health Sciences, Medical College of Rzeszów University, Warzywna 1A, 35-310 Rzeszów, Poland; lidiaiadam.perenc@wp.pl (L.P.); mdruzb@ur.edu.pl (M.D.); agnieszkadepa2@wp.pl (A.G.); 2 Centre for Innovative Research in Medical and Natural Sciences, University of Rzeszow, Warzywna 1a, 35-310 Rzeszow, Poland

**Keywords:** adolescents, children, neurodysfunction, nutritional disorder

## Abstract

A study of the literature shows the lack of data on a comprehensive analysis of eating disorders in children with neurodysfunction, which constitute a clinical subgroup with an increased risk of abnormalities in this area. Therefore, the aim of this study was to determine the relationship between the coexistence of nutritional disorders and diseases or syndromes associated with neurodysfunction based on data collected during hospitalization at a rehabilitation center for children and adolescents. A retrospective analysis was carried out in a group of 327 children and adolescents aged 4–18 years. The study group covered various types of diseases or syndromes involving damage to the central nervous system. A retrospective analysis of baseline data (age, sex, main and additional diagnosis and Body Mass Index—BMI) was performed. Two assessment criteria of nutritional status were taken into account (z-score BMI and other previously published normative values). In the study group, malnutrition was found more frequently (18.0% of the respondents) than obesity (11.3% of the subjects). Hypothyroidism coexisting with malnutrition was identified in the study group (N% = 43.8%, *p* = 0.011) and malnutrition with tetraplegia in the subgroup of spastic cerebral palsy (N% = 34.2 %, *p* = 0.029).

## 1. Introduction

Neurological dysfunctions (DN) in the pediatric population are diagnosed relatively often (e.g., referring to the incidence of cerebral palsy, it amounts to approximately 2 cases/1000 born children for Poland. The approximate frequency is characteristic for all developed countries and has not changed substantially over the last four decades. For comparison, in the United States, it ranges from 2.3 to 3.6/1000) [1,2,3]. Such deviations from average functioning are caused by numerous reasons, including injuries, diseases and syndromes, pregnancy or perinatal complications [3].

Diagnosis of DN usually implies that possibilities of compensatory mechanisms on the part of the nervous system are exhausted [4]. DN significantly impairs the possibilities of intellectual and motor development also limiting rehabilitation prospects, which, in turn, inhibits independent functioning. Thus, the occurrence of DN may constitute a significant barrier hindering recovery of lost skill and acquisition of new skills [3,4]. Patients with neurological dysfunctions may presents wide range of symptoms, which either remain constant or progress due to the progressive nature of some disease entities/syndromes belonging to this group. DS can significantly affect the achievement of individual motor functions (in relation to both fine and gross motor skills), abnormalities in sensory and cognitive functions, intellectual disability, as well as behavioral and psychiatric problems. All of these abnormalities may significantly affect the acquisition of new motor skills or progress in intellectual development and social skills [4,5,6,7,8,9]. Moreover, apart from other coexisting conditions, such as epilepsy, communication difficulties or behavioral problems, neurological disability is also associated with an increased risk of malnutrition [10,11]. The reason for this is the greater demand for nutrients, increased nutrient loss and reduced consumption, which, in turn, may lead to increased disability due to adverse effects on the development of the nervous system, reduction of muscle strength or weakened immunity [11,12].

To the best of our knowledge, no comprehensive analysis is available on nutritional disorders in children with diseases or syndromes involving neurodysfunction as a clinical subgroup at increased risk of disorders of nutritional status. The article regards the subject of ‘eating disorders’ in a new light.

Therefore, the aim of the study was to determine the relationship between the coexistence of nutritional disorders and diseases or syndromes involving neurodysfunction by using basic data collected during admission to the Department of Neurological Rehabilitation of Children and Youth.

## 2. Materials and Methods

Participants: A retrospective analysis was carried out in a group of 327 children and youth admitted to the Department of Neurological Rehabilitation of Children and Youth of St. Hedvig Clinical Provincial Hospital No. 2 in Rzeszów, in the Clinical Regional Rehabilitation and Education Center, between the years 2012 and 2016. Inclusion criteria included age 4–18 (4 years of age was the beginning of the analyzed age range for two reasons: (a) because of the high percentage of children who are under 4 years of age without diagnosis [13,14] (b) because of the methodology adopted in the entire research project [15,16]), informed consent given by both the children (according to Polish law, by children above 16) and their parents and/or legal guardians, congenital defect of the nervous system with at least one neurodysfunction since infancy, and measurements taken (body weight, body height, one hospitalization and complete set of data—diagnosis and anthropometric measurement). For the purposes of this study, retrospectively collected anthropometric parameters, such as body weight and body height, were used. BMI, BMI z-score, and w z-score were calculated from these parameters [16,17]. BMI z-score and w z-score were used for statistical analysis. Exclusion criteria covered age below 4, lack of informed consent given by both the children (according Polish law, by children above 16) and their parents and/or legal guardians, no congenital nervous system disease or neurological syndrome with at least one neurodysfunction since infancy, coexistence of the analyzed congenital nervous system diseases and neurological syndrome with at least one neurodysfunction since the infancy period (e.g., Down’s syndrome and cerebral palsy, neural tube defect and cerebral palsy, phenylketonuria and cerebral palsy), and no measurements taken (body weight, body height, head circumference, other hospitalizations except the selected one and lack of complete set of data—diagnosis and anthropometric measurements). The study was carried out on the entire available sample; all cases (available in a given place, in the analyzed time period) that met the inclusion criteria were included in the analysis—convenience sampling. The age range analyzed in the study results from the uniform reference system used in the study, containing such anthropometric features as body weight (Wt), body height (Ht) and head circumference (HC). Out of 2637 hospitalizations in the years 2012–2016 at the Department of Neurological Rehabilitation of Children and Youth of St. Hedvig Clinical Provincial Hospital No. 2 in Rzeszów, in the Clinical Regional Rehabilitation and Education Center, 327 meeting the eligibility criteria were selected. During the initial analysis, the remaining 2310 were excluded due to failure to meet the inclusion criteria. Retrospective analysis was carried out in a group of 327 children. The mean age of the study group was 9.7 ± 4.3 years (median—9.0 years; the youngest—4 years; and the oldest—18 years). Among 327 children, there were 184 boys (56.27%) and 143 girls (43.73%). Ethical approval was given by the local Bioethics Commission of the University of Rzeszow, and the study was conducted in accordance with applicable Polish law and all methods were performed in this study in accordance with the relevant guidelines and regulations.

Procedures and data analyses: A retrospective analysis of basic data (age, sex, main and additional diagnosis and body mass index—BMI) was collected during the admission procedure of the study group. The statistical methods used were uniform across all articles in the previously published series of which this manuscript is part. We want to maintain the methodological consistency of the cycle. The main and additional diagnoses were established by various specialists (neurologists, geneticists, endocrinologists, etc.) before hospitalization at Clinical Regional Rehabilitation and Education Center and anthropometric measurements were carried out in the Clinical Regional Rehabilitation and Education Center Admission Room according to hospital rules. In the study group, there were various types of diseases or syndromes involving nervous system dysfunction. Their common feature was the congenital character or the occurrence of motor disorders (neurodysfunction) since infancy. Encephalopathy was either present or absent in the analyzed diseases. The subjects were divided into subgroups according to the criteria proposed in the literature (expected presence or absence of encephalopathy, its etiopathogenesis and nature) [18]. Seven subgroups were separated from the entire study group —given the nature and presence of expected encephalopathy [18]: six with diseases/syndromes usually involving encephalopathy, namely progressive in metabolic diseases (E-MD), progressive epilepsy/genetically conditioned epileptic syndromes (EE), non-progressive in neural tube defects (E-NTDs), non-progressive in genetically conditioned diseases (chromosomal aberrations and monogenic diseases, except for neuromuscular diseases) (E-GD), non-progressive toxic (TE) and non-progressive in cerebral palsy (E-CP); and one in the diseases usually without encephalopathy—neuromuscular diseases (NMD). Smaller subgroups were merged into 3 larger ones: with progressive encephalopathy (PE), non-progressive (NPE) and neuromuscular diseases (NMD). Due to the large variety of neural tube defects [19] and significance of further surgical treatment [20], this subgroup was divided into those operated on for myelomeningocele and hydrocephalus, operated on only because of myelomeningocele, and other cases where no surgical treatment was given (see Table 1).

Z-score BMI and criteria used in earlier studies were applied in order to diversify the study group in terms of nutritional status in our study [21,22]. The previously published normative values were administered as the reference system [23]. Table 2 presents selected numerical characteristics of body mass z-score (z-score w) and z-score BMI, such as the arithmetic mean ($\bar{x}$), median (Me), standard deviation (s), minimum (Min) and maximum (Max), and 25th (c25) and 75th (c75) centile. In the study group, the mean and median for z-score w were lower than 0 but higher than −2, and for z-score BMI, they were lower than 0 but higher than −1.

Nutritional status was determined based on the z-score BMI and criteria already used in previous studies (Table 3) [11,15,18,21].

Relationships between the coexistence of nutritional disorders and diseases or syndromes involving neurodysfunction within individual subgroups were sought. Additional diagnoses (symptomatic epilepsy as opposed to genetically determined epileptic syndrome, hypothyroidism) were also included. The dependence analysis was presented in the form of a summary of the number (N) and percentage structure (N%) of answers to selected questions in the compared groups. In the crosstabs, Adjusted Standardized Residuals (ASR) were presented next to the percentages. (ASR) were computed, similar to the analyses presented earlier [15,24]. Values above the limit (>1.96) were considered to indicate increased numbers, and reduced numbers below the limit value (<−1.96). ASR is valuable as it provides additional information about the type of this relationship [25]. To assess to what extent the differentiation of responses between groups reflects a certain regularity prevailing in the entire target population and to what extent it is a matter of random differences, methods of statistical inference were used. The nominal nature of the compared features determines the choice of the chi-square test for independence. To examine the relationship between dependent, qualitative and independent, and quantitative variables, a nominal regression was used. The level of statistical significance was adopted at *p* < 0.05.

## 3. Results

Characteristics regarding basic diagnosis, subgroups, number of respondents (N), their percentage (N%) and abbreviations used are listed in Table 1.

The percentages for the seven separated subgroups are presented as follows: E-MD 2.1%, EE 0.3%, E-NTDs 7.3%, E-GD 7.0%, TE 0.3%, E-CP 73.1% and NMD 9.8%. The division into three previously defined subgroups is as follows: PE, 2.4%; NPE, 87.7%; and NMD, 9.8%.

In the entire study group, cerebral palsy was the most numerous (73.1%). Based on the analyzed diagnoses, the following forms of Hagberg’s cerebral palsy classification were distinguished [18]: spastic, 93.7% (223 people); mixed, 5.0% (12 people); and atactic, 1.7% (4 people). The diskinetic form was absent. Tetraplegia was found in 34.1% (76 people) of spastic cases, in 40.4% with diplegia (90 people) and in 25.6% of children with hemiplegia (57 people). Basic diagnoses were accompanied by additional diagnoses: symptomatic epilepsy, 26.3% (86 people); and hypothyroidism, 4.3% (14 people).

According to classification of nutritional status based on the BMI state of nutrition z-score in division into three categories of nutritional status, normal nutritional status was found in 42.5% of the respondents, with an incidence of malnutrition at 18.0% and underweight at 19.9%. In total, 37.9% of the subjects had body mass deficiency in relation to body height. Overweight occurred in 8.3% and obesity in 11.3%; in total, 19.6% presented excess body weight to body height (Table 4).

The following are statistically insignificant results.

The findings show more/less frequent (%) co-occurrence and no statistically significant correlations between the following:

The first is the five nutritional status categories that were classified based on the z-score BMI and units and syndromes running with neurodysfunction (*p* = 0.292; Cp = 0.548). Malnutrition often occurs with AMC&N (N% = 66.7%, ASR = 2.2) and GLUT1d (N% = 100%, ASR = 2.1). Underweight is often associated with DGS (N% = 100%, ASR = 2.0), HMSN (N% = 50%, ASR = 2.2), rzadko u sasMMC&HCP (N% = 0%, ASR = −2.1). Overweight is often associated with AS (N% = 100%, ASR = 3.3), z LGMD (N% = 26.4%, ASR = −2.2). Obesity often co-occurs with GSD II, MWS, HCP, chromosomal aberration 46XX, del (12) (q24.21q24.23) (N% = 100%, ASR = 2.8) and z DMD (N% = 42.9%, ASR = 2.7), but rarely with CP (N% = 8.8%, ASR = −2.4). (Table 5).

The second is the five nutritional status categories that were classified based on the z-score BMI and classification with regard to etiopathogenesis, and the presence and character encephalopathy (*p* = 0.647; Cp = 0.245). Underweight is often associated with TE (N% = 100%, ASR = 2.0), rarely with NTDs (N% = 4.2%, ASR = −2.0). Obesity rarely co-occurs with CP (N% = 8.8%, ASR = −2.4) (Table 6).

The findings show no more/less frequent (%) co-occurrence and no statistically significant in the following:

The first is the three categories of nutritional status that were classified based on the z-score BMI and units and syndromes running with neurodysfunction (*p* = 0.296; Cp = 0.424) (Table 7).

The second is the three categories of nutritional status that were classified based on the z-score BMI and classification with regard to etiopathogenesis and the presence and character of encephalopathy (*p* = 0.445; Cp = 0.188) (Table 8).

The third is the five nutritional status categories that were classified based on the z-score BMI and classification with regard to presence and character of encephalopathy (*p* = 0.101; Cp = 0.910) (Table 9).

The fourth is the three categories of nutritional status that were classified based on the z-score BMI and classification with regard to presence and character encephalopathy (*p* = 0.753; Cp = 0.076) (Table 10).

The fifth were the five nutritional status categories that were classified based on the z-score BMI and types of CP (*p* = 0.874; Cp = 0.125) (Table 11).

The sixth and final is the three categories of nutritional status that were classified based on the z-score BMI and types of CP (*p* = 0.869; Cp = 0.072) (Table 12).

At a later stage, the relationship between the coexistence of nutritional disorders and diseases or syndromes involving neurodysfunction with individual subgroups, as well as within individual subgroups, was analyzed. We observed the presence of more frequent/rarer co-occurrence and the presence of statistically significant relationships between the following:

The first is the five nutritional status categories that were classified based on the z-score BMI and type of spasticity (Table 13). Malnutrition more often accompanies tetraplegia (N% = 34.2%, ASR = 3.8), and less often diplegia (N% = 11.1%, ASR = −2.8). Normal nutritional status more often accompanies diplegia (N% = 51.1%, ASR = 2.3). The relationship was statistically significant (*p* = 0.029; Cp = 0.267). In the group of children and adolescents with spastic cerebral palsy, malnutrition often occurs with tetraplegia.

The second is the three categories of nutritional status that were classified based on the z-score BMI and the type of spasticity (Table 14). Body mass deficiency is more often accompanied by tetraplegia (N% = 53.9%, ASR = 2.9), less often diplegia (N% = 31.1%, ASR = −2.4). Normal nutritional status is more often accompanied by diplegia (N% = 51.1%, ASR = 2.9). The relationship was statistically significant (*p* = 0.032, Cp = 0.213).

The third is the five categories of nutritional status that were classified based on the z-score BMI and hypothyroidism (Table 15). Normal nutritional status with lack of hypothyroidism is common (N% = 50.0%, ASR = 3.2), and with hypothyroidism is rare (N% = 14.3%, ASR = −2.2). Malnutrition with hypothyroidism is common (N% = 43.8%, ASR = 2.2), and without hypothyroidism is rare (N% = 16.6%, ASR = −3.2). The relationship is statistically significant (*p* = 0.011; Cp = 0.196).

The fourth was the three categories of nutritional status that were classified based on the z-score BMI and hypothyroidism (Table 16). Normal nutritional status with lack of hypothyroidism is common (N% = 43.8%, ASR = 2.2), and with hypothyroidism is rare (N% = 42.9%, ASR = −2.2). Underweight with hypothyroidism is common (N% = 57.1%, ASR = 3.2), and without hypothyroidism is rare (N% = 36.1%, ASR = −3.2). The relationship is statistically significant (*p* = 0.006, Cp = 0.174).

The higher the z-score BMI, the lower was the chance of coexistence of tetraplegia (*p* < 0.001) in the spastic form of cerebral palsy subgroup (95% confidence interval). As the value of the z-score BMI increased, the chance of co-occurrence of tetraplegia decreased (OR = 0.693). The higher the z-score, the lower was the chance of co-occurrence of tetraplegia (*p* < 0.001). As the value of the z-score increased, the chance of co-occurrence of tetraplegia decreased (OR = 0.611) (Table 17A). The higher the z-score BMI, the lower the chance of coexistence of hypothyroidism (*p* = 0.002) in the entire study group (95% confidence interval). As the value of the z-score BMI increased, the chance of coexistence of hypothyroidism (OR = 0.526) decreased. The higher the z-score, the lower the chance of co-occurrence of hypothyroidism (*p* = 0.001). As the value of the z-score w increased, the chance of co-occurrence of hypothyroidism (OR = 0.615) decreased (Table 17B). Hypothyroidism and tetraplegia were not found to coexist.

## 4. Discussion

Children who require special healthcare are often at risk of problems with their body’s poor nutritional status. This risk is associated with the occurrence of many factors, such as parental nutritional status, prenatal nutrition, physiological, medical and/or behavioral factors [26]. A very important factor is motor disorders related to both oromotor dysfunction (chewing and swallowing) and to potentially reduced function in terms of daily activities (the ability to prepare and eat meals independently) [9,27]. The incidence of malnutrition in children with neurological disorders is not fully understood. According to current research, it is estimated that underweight occurs in 1/3 of patients with a significant proportion of them suffering from malnutrition (in the example of cerebral palsy, the estimated frequency is 400/1000) [21,28]. In the case of our study, underweight was found in less than 40% of cases (37.9%), with 18% experiencing malnutrition and 19.9% being underweight, which was almost 10 percentage points higher than in available data in the literature on the subject. Normal nutritional status was presented by 42.5% of the subjects; in turn, excessive body weight, including overweight (8.3%) and obesity (11.3%), was found in 19.6%.

A special subgroup were children with cerebral palsy (CP). This clinical subpopulation, due to the high incidence of these clinical symptoms, has been widely discussed in terms of nutritional status. The conclusions of many studies indicate that this group is exposed to a particularly high risk of malnutrition, and the factors significantly affecting it are the degree of motor disability and oropharyngeal dysfunctions [29] and the duration and severity of neurological disorders [30,31]. Moreover, in the case of our study, the fact of significant influence of neurological disorders on the occurrence of body mass abnormalities in children with CP was confirmed. It was shown that malnutrition is more common in children and adolescents with tetraplegia, rather than with diplegia/hemiplegia. A similar result was obtained for the general category of body mass deficiency (tetraplegia vs. diplegia/hemiplegia). An interesting result, in this respect, was presented by other authors who provided data on the much more frequent occurrence of malnutrition in children with CP with tetraplegia compared to children with diplegia or hemiplegia [21]. Moreover, in another study, researchers analyzed the factors causing weight deficiency in a small group of 61 children with CP and found a relatively high percentage (43%) of children with malnutrition [32]. A cross-sectional study by Turkish Cerebral Palsy Study Group on 1108 pediatric patients with CP showed frequent malnutrition, but the percentages differed depending on the criterion used, ranging from 57.2% (based on clinical evaluation) to 91.3% (based on Waterlow classification of Neyzi height for age (HFA) percentiles), reaching 94.3% (based on Gomez classification of Neyzi weight for age (WFA) percentiles) [33]. The authors of a cross-sectional study from China also presented results of a similar study. By analyzing the 377-person group with CP, they found the incidence of stunting at 42.4%, underweight at 12.7%, thin at 21.5% and excessive body weight at 18.5% [34].

It is also clinically significant that hypothyroidism was identified as an important factor increasing the risk of weight deficiency in our study. This additional diagnosis is relatively common in the case of the Polish population (2–5%). From the clinical point of view, the introduction of this differentiating factor in the analysis was necessary due to the important function of thyroid hormones in the growth process [35,36,37]. Moreover, in a previously published article on growth disorders in children and adolescent affected by syndromes or diseases associated with neurodysfunction, it was stated that, despite the treatment of hypothyroidism in the group of children with neurodysfunction, short stature occurred with high frequency, so its occurrence could mask other causes of short stature [15]. In our own study, there was indeed an interesting relationship from the practical perspective regarding the coexistence of hypothyroidism and weight deficiency.

It is worth emphasizing that we are talking about the co-occurrence of hypothyroidism and malnutrition, or hypothyroidism and body weight deficiency in patients whose symptoms have been manifested since birth or congenital syndromes/diseases, i.e., patients with a damaged nervous system. We also know that hypothyroidism in the examined group of children coexists with short stature. Hypothyroidism is not associated with tetraplegia [15]. These are studies based on anthropometric methods. Other researchers using laboratory methods and hormone determinations indicate that hypothyroidism is commonly thought to be responsible for obesity. However, their causal link is controversial. Excessive (overt) hypothyroidism is associated with small weight gain. An innovative view indicates that changes in thyroid stimulating hormone (TSH) may be secondary to obesity [38]. Thyroid hormones are crucial for the proper development of a child from the early stages of fetal life. They affect the development of the central nervous system in the prenatal period and, up to 3 years of age, regulate the child’s growth process and most metabolic processes [39].

The division of the examined group of children and adolescents with neurodysfunction into subgroups proposed by the authors has already been presented [15,24,40,41]. It is important from a methodological point of view because it allowed to show statistically significant relationships within the specified subgroups, such as, for example, spastic CP [15,24]; NTDs or between subgroups, for example, such as NTDs; NMD out of seven subgroups [24,40]; and NPE out of three subgroups [24]. It was also important to consider the coexistence of hypothyroidism and epilepsy [15,24,40]. The following developmental disorders were examined in the described group and subgroups: microcephaly and macrocephaly [40], relative/absolute microcephaly and relative/absolute macrocephaly [24], short stature and tall stature [40], microsomia and macrosomia [41]. Eating disorders have not been presented so far. Short stature was statistically significantly more frequent in the examined children and adolescents with neurodysfunction and with coexisting hypothyroidism. Another statistically significant relationship could be demonstrated, not in the entire study group, but in separate subgroups. Short stature was statistically significantly more frequent in the case of tetraplegia in the spastic CP subgroup and in patients treated surgically for myelomeningocele and hydrocephalus in the NTDs subgroup [40]. Much more statistically significant relationships were found in the case of developmental disorders of the head (skull) [24,40]. For example, microcephaly was significantly more frequent in the examined children and adolescents and with neurodysfunction and with comorbid hypothyroidism or epilepsy [40]. Macrocephaly was statistically significantly more common in the subgroups: NTDs and NMD, out of seven subgroups [40]. Relative microcephaly was statistically significantly more frequent in the NPE subgroup out of three subgroups [15]. Absolute macrocephaly was statistically significantly more frequent in the subgroups: NTDs and NMD, among seven subgroups [24]. Absolute microcephaly was statistically significantly more frequent in the entire study group in patients with coexisting epilepsy [24]. Contrary to the nutritional disorders discussed in this article, in the previously discussed cases [15,24,40] there are more statistically significant relationships. It is worth noting that patients with tetraplegia in the spastic CP subgroup are burdened with more frequent occurrence of microcephaly [40] and short stature [15], and, as shown by the present results, malnutrition. It seems that further research should focus on the relationship between brain size and body size.

As mentioned earlier, both our research and the results presented by other researchers show that a significant percentage of children with neurological disorders present malnutrition [42]. In the past, the occurrence of low body weight in this group of patients was treated as an unavoidable symptom of the disease resulting from the central nervous system dysfunction [28]. Over the past 30 years, the abnormalities in body weight caused multidisciplinary nutrition programs to be increasingly used for the developmental population with DN. As reported in the literature, they play an important evaluation and therapeutic role, resulting in a reduction in the number of hospitalizations and improved nutrition (in terms of body weight, muscle mass and subcutaneous fat), as well as improved quality of life, in this group of patients [43,44,45,46].

The results of the study indicate that, especially in the group of children and adolescents with syndromes or diseases involving neurodysfunction with low BMI values, screening for hypothyroidism should be obligatory. They indicate that tetraplegia is not the only form coexisting with malnutrition. In addition, tetraplegia and hypothyroidism do not coexist.

## 5. Conclusions

The occurrence of nutritional disorders is observed in the group of children and adolescents with syndromes or diseases involving neurodysfunction. In the studied group, malnutrition coexisting with hypothyroidism was found, and in the subgroup with spastic form of cerebral palsy, the coexistence of malnutrition with tetraplegia was observed. In the future, a group of children and adolescents with syndromes or diseases with neurodysfunction should be covered by diagnostic and therapeutic intervention focused on malnutrition in conjunction with evaluation of its effectiveness.

## 6. Limitations

The study was retrospective, and the data from medical records were scarce. From a clinical point of view, other factors that significantly affect the differentiation of body weight categories, such as intellectual and physical disability, dysphagia severity, feeding method, type of food consumed and hormonal balance, should be taken into account in future prospective studies.

## Figures and Tables

**Table 1:** Characteristics of the study group.

Study Group—Recognition, Division into Subgroups, Abbreviation								
Units and syndromes running with neurodysfunction (Main recognition)			Classification with regard to etiopathogenesis, presence and character encephalopathy			Classification with regard to presence and character encephalopathy		
	N	N%		N	N%		N	N%
NBIA–MPAN, Neurodegeneration with Brain Iron Accumulation–Mitochondrial Protein Associated Neurodegeneration	2	0.6	E-MD, encephalopathy in metabolic disorder	7	2.1	PE, progressive encephalopathy	8	2.4
GSD II, Pompe’s disease	1	0.3						
LCHAD, long-chain 3-hydroxyacyl-coenzyme A dehydrogenase deficiency	1	0.3						
SLO, Smith–Lemli–Opitz syndrome	1	0.3						
GLUT1d, glucose transporter 1 deficiency	1	0.3						
NKH, nonketotic hyperglycinemia	1	0.3						
SMEI, Dravet’s syndrome	1	0.3	EE, epileptic encephalopathy	1	0.3			
sasMMC&HCP, state after surgery lumbar myelomeningocele and hydrocephalus	17	5.2	E-NTDs, encephalopathy in neural tube defects	24	7.3	NPE, non-progressive encephalopathy	287	87.7
sasMMC, state after surgery lumbar myelomeningocele	3	0.9						
sasMM, state after surgery parietoocipital meningocele	1	0.3						
ACM, Arnold–Chiari malformation	2	0.6						
HCP, isolated hydrocephalus	1	0.3						
DS, Down’s syndrome	11	3.4	E-GD, encephalopathy in genetic disorders	23	7.0			
ES, Edwards syndrome	1	0.3						
PMS, Phelan–McDermid syndrome	2	0.6						
MWS, Mowat–Wilson syndrome	1	0.3						
AS, Angelman syndrome	1	0.3						
DGS, Di George syndrome	1	0.3						
46XY,del(X)(q24)	1	0.3						
CdLS, Cornelia de Lange syndrome	1	0.3						
SDS, Schwachman–Diamond syndrome	1	0.3						
PWS, Prader–Willi syndrome	1	0.3						
46XX,add(2)(q25)	1	0.3						
46XX,del(12)(q24.21q24.23)	1	0.3						
FAS, fetal alcohol syndrome	1	0.3	TE, toxic encephalopathy	1	0.3			
CP, cerebral palsy	239	73.1	E-CP encephalopathy in cerebral palsy	239	73.1			
HMSN, hereditary motor and sensory polyneuropathy	8	2.4	NMD, neuromuscular disorders	32	9.8	NMD, neuromuscular disorders	32	9.8
LGMD, muscular dystrophy limb-girdle	7	2.1						
BMD, Becker’s muscular dystrophy	3	0.9						
DMD, Duchenne muscular dystrophy	7	2.1						
TD, Thomsen disease	1	0.3						
AMC&N arthrogryposis multiplex congenita with neuropathy	3	0.9						
CM, congenital myopathy	1	0.3						
SMA, spinal muscular atrophy	2	0.6						
In total	327	100	In total	327	100	In total	327	100

**Table 2:** Numerical characteristics of the z-score, z-score w and z-score BMI.

Z-Score	*N*	$\bar{x}$	Me	*S*	*c*_25_	*c*_75_	Min	Max
z-score w	327	−0.78	−1.05	1.98	−1.90	0.17	−10.52	8.75
z-score BMI		−0.33	−0.64	1.76	−1.45	0.46	−4.20	6.98

Arithmetic mean ($\bar{x}$), median (Me), standard deviation (s), smallest (Min) and largest value (Max), 25th centile (*c*_25_) and 75th (*c*_75_).

**Table 3:** Classification of nutritional status based on the z-score BMI.

Malnutrition	z-score BMI < −1.64	Body mass deficiency in relation to height	z-score BMI < −1
Underweight	−1.64 ≥ z-score BMI < −1		
Correct state of nutrition	−1 ≥ z-score BMI ≤ 1	Normal nutritional status	−1 ≥ z-score BMI ≤ 1
Overweight	1 > z-score BMI ≤ 1.64	Excess body weight in relation to height	z-score BMI > 1
Obesity	z-score BMI > 1.64		

**Table 4:** The state of nutrition—the frequency of occurrence of each category.

Five categories of nutritional status	N	N%	Three categories of nutritional status	N	N%
Malnutrition	59	18	Body mass deficiency in relation to height	124	37.9
Underweight	65	19.9			
Correct state of nutrition	139	42.5	Normal nutritional status	139	42.5
Overweight	27	8.3	Excess body weight in relation to height	64	19.6
Obesity	37	11.3			

**Table 5:** Relationship between five categories of nutritional status classified based on the BMI z-score and units and syndromes running with neurodysfunction.

Units and Syndromes Running with Neurodysfunction (Main Recognition)	Five Categories of Nutritional Status Classified Based on the Z-Score BMI (*p* = 0.292; Cp = 0.548)										In Total
	Malnutrition		Underweight		Correct State		Overweight		Obesity		
	N (N%)	ASR	N (N%)	ASR	N (N%)	ASR	N (N%)	ASR	N (N%)	ASR	N
NBIA–MPAN	0	−0.7	1 (50%)	1.1	1 (50%)	0.2	0	−0.4	0	−0.5	2
GSD II	0	−0.5	0	−0.5	0	−0.9	0	−0.3	1 (100%)	2.8	1
LCHAD	0	−0.5	0	−0.5	1 (100%)	1.2	0	−0.3	0	−0.4	1
SLO	0	−0.5	0	−0.5	1 (100%)	1.2	0	−0.3	0	−0.4	1
GLUT1d	1 (100%)	2.1	0	−0.5	0	−0.9	0	−0.3	0	−0.4	1
NKH	1 (100%)	2.1	0	−0.5	0	−0.9	0	−0.3	0	−0.4	1
SMEI	0	−0.5	0	−0.5	1 (100%)	1.2	0	−0.3	0	−0.4	1
sasMMC&HCP	2 (11.8%)	−0.7	0	−2.1	9 (52.9%)	0.9	2 (11.8%)	0.5	4 (23.5%)	1.6	17
sasMMC	0	−0.8	0	−0.9	2 (66.7%)	0.9	1 (33.3%)	1.6	0	−0.6	3
sasMM	0	−0.5	1 (100%)	2.0	0	−0.9	0	−0.3	0	−0.4	1
ACM	1 (50%)	1.2	0	−0.7	1 (50%)	0.2	0	−0.4	0	−0.5	2
HCP	0	−0.5	0	−0.5	0	−0.9	0	−0.3	1 (100%)	2.8	1
DS	1 (9.1%)	−0.8	3 (27.3%)	0.6	6 (54.5%)	0.8	0	−1.0	1 (9.1%)	−0.2	11
ES	0	−0.5	1 (100%)	2.0	0	−0.9	0	−0.3	0	−0.4	1
PMS	0	−0.7	1 (50%)	1.1	0	−1.2	0	−0.4	1 (50%)	1.7	2
MWS	0	−0.5	0	−0.5	0	−0.9	0	−0.3	1 (100%)	2.8	1
AS	0	−0.5	0	−0.5	0	−0.9	1 (100%)	3.3	0	−0.4	1
DGS	0	−0.5	1 (100%)	2.0	0	−0.9	0	−0.3	0	−0.4	1
46.XY.del(X)(q24)	0	−0.5	0	−0.5	1 (100%)	1.2	0	−0.3	0	−0.4	1
CdLS	0	−0.5	0	−0.5	1 (100%)	1.2	0	−0.3	0	−0.4	1
SDS	0	−0.5	0	−0.5	1 (100%)	1.2	0	−0.3	0	−0.4	1
PWS	0	−0.5	0	−0.5	1 (100%)	1.2	0	−0.3	0	−0.4	1
46 XX. add(2)(q25)	0	−0.5	0	−0.5	1 (100%)	1.2	0	−0.3	0	−0.4	1
46XX. del (12) (q24.21q24.23)	0	−0.5	0	−0.5	0	−0.9	0	−0.3	1 (100%)	2.8	1
FAS	0	−0.5	1 (100%)	2.0	0	−0.9	0	−0.3	0	−0.4	1
CP	48	1.6	50 (20.9%)	0.8	100 (41.8%)	−0.4	20 (8.4%)	0.1	21 (8.8%)	−2.4	239
HMSN	0	−1.3	4 (50%)	2.2	2 (25%)	−1.0	1 (12.5%)	0.4	1 (12.5%)	0.1	8
LGMD	1 (14.3%)	−0.3	0	−1.3	3 (42.9%)	0.0	2 (28.6%)	2.0	1 (14.3%)	−0.3	7
BMD	0	−0.8	1 (33.3%)	0.6	2 (66.7%)	0.9	0	−0.5	0	−0.6	3
DMD	2 (28.6%)	0.7	1 (14.3%)	−0.4	1 (14.3%)	−1.5	0	−0.8	3 (42.9%)	2.7	7
TD	0	−0.5	0	−0.5	0	−0.9	0	−0.3	1 (100%)	2.8	1
AMC&N	2 (66.7%)	2.2	0	−0.9	1 (33.3%)	−0.3	0	−0.5	0	−0.6	3
CM	0	−0.5	0	−0.5	1 (100%)	1.2	0	−0.3	0	−0.4	1
SMA	0	−0.7	0	−0.7	2 (100%)	1.6	0	−0.4	0	−0.5	2
In total	59 (18%)		65 (19.9%)		139 (42.5%)		27 (8.3%)		37 (11.3%)		327 (100%)

N—numbers of patients, %—percent, *p*—probability value calculated by chi-square test of independence, Cp—Pearson’s Contingency Coefficient C, Cp ≥ 0, values distant from 0 reflect some relationship; values approaching 1 correspond to a perfect association, ASR—Adjusted Standardized Residuals, values >1.96 reflect a greater number, and those below <−1.96 correspond to a smaller number than a random distribution.

**Table 6:** Relationship between five categories of nutritional status classified based on the BMI z-score and classification with regard to etiopathogenesis, presence and character encephalopathy.

Classification with Regard to Etiopathogenesis, Presence and Character Encephalopathy	Five Categories of Nutritional Status Classified Based on the Z-Score BMI (*p* = 0.647; Cp = 0.245)										In Total
	Malnutrition		Underweight		Correct State		Overweight		Obesity		
	N (N%)	ASR	N (N%)	ASR	N (N%)	ASR	N (N%)	ASR	N (N%)	ASR	N
MD	2 (28.6%)	0.7	1 (14.3%)	−0.4	3 (42.9%)	0.0	0	−0.8	1 (14.3%)	0.3	7
EE	0	−0.5	0	−0.5	1 (100%)	1.2	0	−0.3	0	−0.4	1
NTDs	3 (12.5%)	−0.7	1 (4.2%)	−2.0	12 (50%)	0.8	3 (12.5%)	0.8	5 (20.8%)	1.5	24
GD	1 (4.3%)	−1.8	6 (26.1%)	0.8	11 (47.8%)	0.5	1 (4.3%)	−0.7	4 (17.4%)	1.0	23
TE	0	−0.5	1 (100%)	2.0	0	−0.9	0	−0.3	0	−0.4	1
CP	48 (20.1%)	1.6	50 (20.9%)	0.8	100 (41.8%)	−0.4	20 (8.4%)	0.1	21 (8.8%)	−2.4	239
NMD	5 (15.6%)	−0.4	6 (18.8%)	−0.2	12 (37.5%)	−0.6	3 (9.4%)	0.2	6 (18.8%)	1.4	32
In total	59 (18%)		65 (19.9%)		139 (42.5%)		27 (8.3%)		37 (11.3%)		327 (100%)

**Table 7:** Relationship between three categories of nutritional status classified based on the BMI z-score and units and syndromes running with neurodysfunction.

Units and Syndromes Running with Neurodysfunction (Main Recognition)	Three Categories of Nutritional Status Classified Based on the Z-Score BMI (*p* = 0.296; Cp = 0.424)						In Total
	Body Mass Deficiency		Correct State		Excess Body Weight		
	N (N%)	ASR	N (N%)	ASR	N (N%)	ASR	N
NBIA-MPAN	1 (50%)	0.4	1 (50%)	0.2	0	−0.7	2
GSD II	0	−0.8	0	−0.9	1 (100%)	2.0	1
LCHAD	0	−0.8	1 (100%)	1.2	0	−0.5	1
SLO	0	−0.8	1 (100%)	1.2	0	−0.5	1
GLUT1d	1 (100%)	1.3	0	−0.9	0	−0.5	1
NKH	1 (100%)	1.3	0	−0.9	0	−0.5	1
SMEI	0	−0.8	1 (100%)	1.2	0	−0.5	1
sasMMC&HCP	2 (11.8%)	−2.3	9 (52.9%)	0.9	6 (35.3%)	1.7	17
sasMMC	0	−1.4	2 (66.7%)	0.9	1 (33.3%)	0.6	3
sasMM	1 (100%)	1.3	0	−0.9	0	−0.5	1
ACM	1 (50%)	0.4	1 (50%)	0.2	0	−0.7	2
HCP	0	−0.8	0	−0.9	1 (100%)	2.0	1
DS	4 (36.4%)	−0.1	6 (54.5%)	0.8	1 (9.1%)	−0.9	11
ES	1 (100%)	1.3	0	−0.9	0	−0.5	1
PMS	1 (50%)	0.4	0	−1.2	1 (50%)	1.1	2
MWS	0	−0.8	0	−0.9	1 (100%)	2.0	1
AS	0	−0.8	0	−0.9	1 (100%)	2.0	1
DGS	1 (100%)	1.3	0	−0.9	0	−0.5	1
46.XY.del(X)(q24)	0	−0.8	1 (100%)	1.2	0	−0.5	1
CdLS	0	−0.8	1 (100%)	1.2	0	−0.5	1
SDS	0	−0.8	1 (100%)	1.2	0	−0.5	1
PWS	0	−0.8	1 (100%)	1.2	0	−0.5	1
46 XX. add(2)(q25)	0	−0.8	1 (100%)	1.2	0	−0.5	1
46XX. del (12) (q24.21q24.23)	0	−0.8	0	−0.9	1 (100%)	2.0	1
FAS	1 (100%)	1.3	0	−0.9	0	−0.5	1
CP	98 (41%)	1.9	100 (41.8%)	−0.4	41 (17.2%)	−1.8	239
HMSN	4 (50%)	0.7	2 (25%)	−1.0	2 (25%)	0.4	8
LGMD	1 (14.3%)	−1.3	3 (42.9%)	0.0	3 (42.9%)	1.6	7
BMD	1 (33.3%)	−0.2	2 (66.7%)	0.9	0	−0.9	3
DMD	3 (42.9%)	0.3	1 (14.3%)	−1.5	3 (42.9%)	1.6	7
TD	0	−0.8	0	−0.9	1 (100%)	2.0	1
AMC&N	2 (66.7%)	1.0	1 (33.3%)	−0.3	0	−0.9	3
CM	0	−0.8	1 (100%)	1.2	0	−0.5	1
SMA	0	−1.1	2 (100%)	1.6	0	−0.7	2
In total	124 (37.9%)		139 (42.5%)		37 (11.3%)		327 (100%)

**Table 8:** Relationship between three categories of nutritional status classified based on the BMI z-score and classification with regard to etiopathogenesis, presence and character encephalopathy.

Classification with Regard to Etiopathogenesis, Presence and Character Encephalopathy	Three Categories of Nutritional Status Classified Based on the Z-Score BMI (*p* = 0.445; Cp = 0.188)						In Total
	Body Mass Deficiency		Correct State		Excess Body Weight		
	N (N%)	ASR	N (N%)	ASR	N (N%)	ASR	N
MD	3 (42.9%)	0.3	3 (42.9%)	0.0	1 (14.3%)	−0.4	7
EE	0	−0.8	1 (100%)	1.2	0	−0.5	1
NTDs	4 (16.7%)	−2.2	12 (50%)	0.8	8 (33.3%)	1.8	24
GD	7 (30.4%)	−0.8	11 (47.8%)	0.5	5 (21.7%)	0.3	23
TE	1 (100%)	1.3	0	−0.9	0	−0.5	1
CP	98 (41%)	1.9	100 (41.8%)	−0.4	41 (17.2%)	−1.8	239
NMD	11 (34.4%)	−0.4	12 (37.5%)	−0.6	9 (28.1%)	1.3	32
In total	124 (37.9%)		139 (42.5%)		64 (19.6%)		327 (100%)

**Table 9:** Relationship between five categories of nutritional status classified based on the BMI z-score and classification with regard to presence and character encephalopathy.

Classification with Regard to Presence and Character Encephalopathy	Five Categories of Nutritional Status Classified Based on the Z-Score BMI (*p* = 0.101; Cp = 0.910)										In Total
	Malnutrition		Underweight		Correct State		Overweight		Obesity		
	N (N%)	ASR	N (N%)	ASR	N (N%)	ASR	N (N%)	ASR	N (N%)	ASR	N
PE	2 (2%)	0.5	1 (12.5%)	−0.5	4 (50%)	0.4	0	−0.9	1 (12.5%)	0.1	8
NPE	52 (18.1%)	0.1	58 (20.2%)	0.4	123 (42.9%)	0.3	24 (8.4%)	0.2	30 (10.5%)	−1.3	287
NMD	5 (15.6%)	−0.4	6 (18.8%)	−0.2	12 (37.5%)	−0.6	3 (9.4%)	0.2	6 (18.8%)	1.4	32
In total	59 (18%)		65 (19.9%)		139 (42.5%)		27 (8.3%)		37 (11.3%)		327 (100%)

**Table 10:** Relationship between three categories of nutritional status classified based on the BMI z-score and classification with regard to presence and character encephalopathy.

Classification with Regard to Presence and Character Encephalopathy	Three Categories of Nutritional Status Classified Based on the Z-Score BMI (*p* = 0.753; Cp = 0.076)						In Total
	Body Mass Deficiency		Correct State		Excess Body Weight		
	N (N%)	ASR	N (N%)	ASR	N (N%)	ASR	N
PE	3 (37.5%)	0.0	4 (50%)	0.4	1 (12.5%)	−0.5	8
NPE	110 (38.3%)	0.4	123 (42.9%)	0.3	54 (18.8%)	0.3	287
NMD	11 (34.4%)	−0.4	12 (37.5%)	−0.6	9 (28.1%)	1.3	32
In total	124 (37.9%)		139 (42.5%)		64 (19.6%)		327 (100%)

**Table 11:** Relationship between five categories of nutritional status classified based on the BMI z-score and types of CP.

Types of CP	Five Categories of Nutritional Status Classified Based on the Z-Score BMI (*p* = 0.874; Cp = 0.125)										In Total
	Malnutrition		Underweight		Correct State		Overweight		Obesity		
	N (N%)	ASR	N (N%)	ASR	N (N%)	ASR	N (N%)	ASR	N (N%)	ASR	N
Spastic type	45 (20.2%)	0.1	46 (20.6%)	−0.4	93 (41.7%)	−0.2	20 (9%)	1.3	19 (8.5%)	−0.5	223
Atactic type	0	−1.0	1 (25%)	0.2	2 (50%)	0.3	0	−0.6	1 (25%)	1.2	4
Mixed type	3 (25%)	0.4	3 (25%)	0.4	5 (41.7%)	0.0	0	−1.1	1 (8.3%)	−0.1	12
In total	48 (20.1%)		50 (20.9%)		100 (41.8%)		20 (8.4%)		21 (8.8%)		327 (100%)

**Table 12:** Relationship between three categories of nutritional status classified based on the BMI z-score and types of CP.

Types of CP	Three Categories of Nutritional Status Classified Based on the Z-Score BMI (*p* = 0.869; Cp = 0.072)						In Total
	Body Mass Deficiency		Correct State		Excess Body Weight		
	N (N%)	ASR	N (N%)	ASR	N (N%)	ASR	N
Spastic type	91 (40.8%)	−0.2	93 (41.7%)	−0.2	39 (17.5%)	0.5	223
Atactic type	1 (25%)	−0.7	2 (50%)	0.3	1 (25%)	0.4	4
Mixed type	6 (50%)	0.7	5 (41.7%)	0.0	1 (8.3%)	−0.8	12
In total	98 (41%)		100 (41.8%)		41 (17.2%)		239 (100%)

**Table 13:** Relationship between five categories of nutritional status and type of spasticity.

Type of Spasticity	Five Categories of Nutritional Status Classified Based on the Z-Score BMI (*p* = 0.029; Cp = 0.267)										
	Malnutrition		Underweight		Correct State		Overweight		Obesity		In Total
	N (N%)	ASR	N (N%)	ASR	N (N%)	ASR	N (N%)	ASR	N (N%)	ASR	N
Diplegia	10 (11.1%)	−2.8	18 (20%)	−0.2	46 (51.1%)	2.3	8 (8.9%)	0.0	8 (8.9%)	0.2	57
Hemiplegia	9 (15.8%)	−1.0	13 (22.8%)	0.5	22 (38.6%)	0.5	7 (12.3%)	1.0	6 (10.5%)	0.6	90
Tetraplegia	26 (34.2%)	3.8	15 (19.7%)	−0.2	25 (32.9%)	−1.9	5 (6.6%)	−0.9	5 (6.6%)	−0.7	76
In total	45 (20.2%)		46 (20.6%)		93 (41.7%)		20 (9.0%)		19 (8.5%)		223 (100%)

**Table 14:** Relationship between three categories of nutritional status and type of spasticity.

Type of Spasticity	Three Categories of Nutritional Status Classified Based on the Z-Score BMI (*p* = 0.032; Cp = 0.213)						
	Body Mass Deficiency		Correct State		Excess Body Weight		In Total
	N (N%)	ASR	N (N%)	ASR	N (N%)	ASR	N
Diplegia	28 (31.1%)	−2.4	46 (51.1%)	2.3	16 (17.8%)	0.1	90
Hemiplegia	22 (38.6%)	−0.4	22 (38.6%)	0.5	13 (22.8%)	1.2	57
Tetraplegia	41 (53.9%)	2.9	25 (32.9%)	−1.9	10 (13.2%)	−1.2	76
In total	91 (40.8%)		93 (41.7%)		39 (17.5%)		233 (100%)

**Table 15:** Relationship between five categories of nutritional status and accompanying recognition hypothyroidism.

Hypothyroidism	Five Categories of Nutritional Status Classified Based on the Z-Score BMI (*p* = 0.011; Cp = 0.196))										
	Malnutrition		Underweight		Correct State		Overweight		Obesity		In Total
	N (N%)	ASR	N (N%)	ASR	N (N%)	ASR	N (N%)	ASR	N (N%)	ASR	N
Present	7 (50%)	3.2	4 (28.6%)	0.8	2 (14.3%)	−2.2	0 (0%)	−1.1	1 (7.1%)	−0.5	14
Lack	52 (16.6%)	−3.2	61 (19.5%)	−0.8	137 (43.8%)	2.2	27 (8.6%)	1.1	36 (11.5%)	0.5	313
In total	59 (18.0%)		65 (19.9%)		139 (42.5%)		27 (8.3%)		37 (11.3%)		327 (100%)

**Table 16:** Relationship between three categories of nutritional status and accompanying recognition hypothyroidism.

Hypothyroidism	Three Categories of Nutritional Status Classified Based on the Z-Score BMI (*p* = 0.006; Cp = 0.174)						
	Body Mass Deficiency		Correct State		Excess Body Weight		In Total
	N (N%)	ASR	N (N%)	ASR	N (N%)	ASR	N
Present	8 (57.1%)	3.2	6 (42.9%)	−2.2	1 (7.1%)	−1.2	14
Lack	113 (36.1%)	−3.2	137 (43.8%)	2.2	63 (20.1%)	1.2	313
In total	124 (37.9%)		139 (42.5%)		64 (19.6%)		327 (100.0%)

**Table 17:** Relationship between type of spasticity/hypothyroidism and z-score w, z-score BMI.

** (A) Relationship between type of spasticity and z-score w, z-score BMI **				
**Nominal Regression**		**Quantitative Dependent** **Z-Score w**	**Quantitative Dependent** **Z-Score BMI**	
Qualitative dependent Type of spasticity	Tetraplegia (34.1%)	<0.001	<0.001	*p*
		0.611 0.491–0.761	0.693 0.564–0.851	OR
	Others: diplegia, hemiplegia (65.9%)	Reference group		
**(B) Relationship between hypothyroidism and z-score w, z-score BMI**				
**Nominal Regression**		**Quantitative Dependent** **Z-Score w**	**Quantitative Dependent** **Z-Score BMI**	
Qualitative dependent Accompanying recognition	Present of hypothyroidism (4.3%)	0.001	0.002	*p*
		0.615 0.458–0.825	0.526 0.348–0.795	OR
	Lack of hypothyroidism (95.7%)	Reference group		

*p*—test probability value calculated by the test of independence chi-square; OR—odds ratio (95% confidence interval).
